# *Bifidobacterium breve* with α-linolenic acid alters the composition, distribution and transcription factor activity associated with metabolism and absorption of fat

**DOI:** 10.1038/srep43300

**Published:** 2017-03-07

**Authors:** Elaine Patterson, Rebecca Wall, Sara Lisai, R. Paul Ross, Timothy G. Dinan, John F. Cryan, Gerald F. Fitzgerald, Sebastiano Banni, Eamonn M. Quigley, Fergus Shanahan, Catherine Stanton

**Affiliations:** 1 APC Microbiome Institute, Biosciences Building, University College Cork, Cork, Ireland; 2 Teagasc Food Research Centre, Food Biosciences Department, Moorepark, Fermoy, Cork, Ireland; 3 Department of Biomedical Sciences, University of Cagliari, Monserrato, CA 09042, Italy; 4 Department of Psychiatry and Neurobehavioural Science, University College Cork, Cork, Ireland; 5 Department of Anatomy and Neuroscience, University College Cork, Cork, Ireland; 6 School of Microbiology, University College Cork, Cork, Ireland

## Abstract

This study focused on the mechanisms that fatty acid conjugating strains - *Bifidobacterium breve* NCIMB 702258 and *Bifidobacterium breve* DPC 6330 - influence lipid metabolism when ingested with α-linolenic acid (ALA) enriched diet. Four groups of BALB/c mice received ALA enriched diet (3% (w/w)) either alone or in combination with *B. breve* NCIMB 702258 or *B. breve* DPC 6330 (10^9^ CFU/day) or unsupplemented control diet for six weeks. The overall *n*-3 PUFA score was increased in all groups receiving the ALA enriched diet. Hepatic peroxisomal beta oxidation increased following supplementation of the ALA enriched diet with *B. breve (P* < 0.05) and so the ability of the strains to produce *c*9*t*11 conjuga*t*ed linoleic acid (CLA) was identified in adipose tissue. Furthermore, a strain specific effect of *B. breve* NCIMB 702258 was found on the endocannabinoid system (ECS). Liver triglycerides (TAG) were reduced following ALA supplementation, compared with unsupplemented controls (*P* < 0.01) while intervention with *B. breve* further reduced liver TAG (*P* < 0.01), compared with the ALA enriched control. These data indicate that the interactions of the gut microbiota with fatty acid metabolism directly affect host health by modulating *n*-3 PUFA score and the ECS.

Several studies support a communication axis between the gut microbiota and adipose tissue, which influence the development of metabolic alterations associated with obesity. Conventionally raised mice have long been known to have >40% more fat mass than their isocaloric-fed germ-free counterparts^1^ and more recently, it was shown that transplantation of the gut microbiota from obese mice to germ-free mice resulted in a greater increase in total body fat mass accumulation compared with those colonised with gut microbiota isolated from lean donors^2^. The resistance of germ-free mice to high fat diet induced body weight gain and fat mass accumulation has also been described, suggesting that the gut microbiota may promote fat storage^3^.

Furthermore, the gut microbiota play a role in the low grade inflammation associated with obesity and related metabolic disorders^4^,^5^,^6^,^7^,^8^. Gut microbiota-derived lipopolysaccharide (LPS) may represent a cause for the onset and progression of inflammation (metabolic endotoxemia) associated with insulin resistance, type 2 diabetes and high fat diet induced increased fat mass. The endocannabinoid system (ECS) interconnects obesity, inflammation and the gut microbiota in the regulation of energy homeostasis, appetite^9^ and gut barrier function^10^ *via* the microbiota-gut-brain axis. It has been demonstrated that the gut microbiota control ECS activity in the intestine and adipose tissue^11^,^12^ through gut microbial regulation of CB_1_ receptor expression^11^. The ECS system is composed mainly of the bioactive lipids anandamide (AEA; an N-arachidonoylethanolamine), 2 arachidonoylgycerol (2-AG) (synthesised locally in the gastrointestinal tract), the proteins that regulate their production/degradation and the cannabinoid receptors CB_1_ and CB_2_, through which they signal. Obesity is associated with an increase in ECS tone and an altered expression of CB_1_. An increase in endogenous production and content of AEA in the colon and both visceral and subcutaneous adipose tissue^11^,^12^ was blunted by prebiotic treatment^11^. By altering gut microbiota composition through prebiotic feeding, colonic CB_1_ mRNA expression is reduced and antibiotic treatment also decreased the expression of the CB_1_ receptor in the colon. These results correlated with reduced colonic AEA (endogenous CB_1_ ligand), increased fatty acid amine hydrolase (the main enzyme in degradation of AEA) and reduced plasma LPS^11^. Therefore, the gut microbiota, the innate immune system and the ECS interact in the development of obesity and related disorders.

We previously demonstrated that fatty acid composition of the host is influenced by microbial metabolism in the gut and that the effects were strain-specific. *B. breve* NCIMB 702258 and *B. breve* DPC 6330 are efficient producers of conjugated linoleic acid (CLA), converting up to 65% and 76%, respectively, of linoleic acid to *cis-*9, *trans-*11 CLA when grown in 0.5 mg/mL linoleic acid *in vitro*^13^,^14^. We have also demonstrated the ability of these two strains to produce other conjugated fatty acids, including conjugated α-linolenic acid (CNLA), conjugated γ-linoleic acid and conjugated stearidonic acid^15^. Dietary supplementation with *B. breve* NCIMB 702258 and *B. breve* DPC 6330 positively influenced tissue fatty acid profiles in different animal species and models, while also influencing host intestinal microbiota composition^16^,^17^,^18^,^19^.

These data suggest that dietary supplementation with a commensal bacterium can significantly influence health through the production of bioactive conjugated fatty acids and by increasing tissue concentrations of bioactive long-chain (LC) members of the *n*-3 PUFA family (eicosapentaenoic acid (EPA; C20:5), docosapentaenoic acid (C22:5) and docosahexaenoic acid (DHA; C22:6). While CLA isomers have demonstrated anti-atherosclerotic^20^ and anti-diabetogenic^21^,^22^ properties, both CLA and CNLA isomers have exhibited anti-carcinogenic^23^,^24^,^25^,^26^,^27^ and anti-obesogenic^28^,^29^,^30^,^31^,^32^,^33^ properties. Bioactive EPA, docosapentaenoic acid and DHA can partly inhibit a number of aspects of inflammation including leukocyte chemotaxis, adhesion molecule expression and leukocyte–endothelial adhesive interactions, production of *n-6* PUFA derived eicosanoids from arachidonic acid, production of inflammatory cytokines and T-helper 1 lymphocyte reactivity, extensively reviewed^34^. Thus, interactions between resident gut microbes and dietary derived fatty acids with implications for health have been described^35^,^36^,^37^.

While optimal dietary intakes of *n*-6:*n*-3 PUFA should be in the ratio of ~1–4:1; recent shifts in dietary PUFA consumption have established this ratio as ~20:1 in Western diets, favouring *n*-6 PUFA metabolism^38^. Considering that *n*-6 PUFA are precursors of inflammatory eicosanoids (2-series prostaglandins, 2-series thromboxanes and 4-series leukotrienes), it is unsurprising that the incidence of diseases strongly associated with inflammatory processes such as cardiovascular disease (CVD), obesity, inflammatory bowel disease (IBD) and cancer have all been linked to high intakes of *n*-6:*n*-3 PUFA^34^. In contrast, eicosanoids derived from *n*-3 PUFA (3-series prostaglandins, 3-series thromboxanes and 5-series leukotrienes) are broadly anti-inflammatory, however, the benefits of ALA consumption on cardiovascular health, immune regulation and inflammation have been described as moderately positive^39^.

The elongation and desaturation of PUFAs, primarily occurs in the liver^38^, but ALA is considered a poor precursor for DHA due to the efficiency to undergo beta-oxidation in the mitochondria and because biosynthesis of DHA requires a crucial step in the peroxisome for partial beta-oxidation^40^. However, in specific dietary supplementation, ALA increased tissue concentrations of EPA and DHA^17^,^41^,^42^, in fact, we have previously demonstrated that supplementing an ALA enriched diet with *B. breve* NCIMB 702258 and *B. breve* DPC 6330 increased tissue concentrations of EPA, docosapentaenoic acid and DHA, compared with dietary ALA alone^17^,^19^, favouring DHA biosynthesis. Flaxseed has thus emerged as an important functional food ingredient, as it is one of the richest sources of ALA (1 tablespoon of flaxseed oil contains ~8 g ALA)^43^.

Therefore, the aim of this study was to investigate the impact of dietary ALA enrichment with or without fatty acid conjugating microbial supplementation on fat composition and distribution and to investigate the mechanisms through which commensal gut microbes may alter lipid metabolism.

## Results

### Survival and transit of *B. breve* DPC 6330 and *B. breve* NCIMB 702258 in Balb/c mice

Quantification of the numbers of administered *B. breve* strains in the feces of mice confirmed gastrointestinal transit and survival. Stool recovery of *B. breve* NCIMB 702258 and *B. breve* DPC 6330 were approximately 1 × 10^7^ CFU/g feces and 5 × 10^6^ CFU/g feces, respectively, after 1 week of dietary supplementation and remained at similar numbers at weeks 2 and 4. At week 6, there was a decline in the numbers of excreted *B. breve* strains, with stool recovery of *B. breve* NCIMB 702258 and *B. breve* DPC 6330 being 9 × 10^5^ CFU/g feces and 8 × 10^5^ CFU/g feces, respectively. Mice that received the bacterial strains had similar CFU/g after the treatment and importantly, *B. breve* was not isolated from any of the unsupplemented mice or mice receiving ALA alone.

### Dietary supplementation with *B. breve* NCIMB 702258 and *B. breve* DPC 6330 reduced liver TAG levels

No differences in food intake, body weight gain, visceral fat mass or serum TAG levels were observed between the groups (Table 1). Analysis of total liver TAG showed that ALA supplementation, either alone (**ALA-CON**) or in combination with either *B. breve* strain (**ALA + NCIMB 702258/ALA + DPC 6330**) resulted in reduced liver TAG levels, compared with the unsupplemented control (**CON**) (*P* < 0.01; Table 1). Furthermore, ALA + NCIMB 702258 and ALA + DPC 6330 exhibited reduced liver TAG, compared with ALA-CON (*P* < 0.01; Table 1).

### Effect of dietary enrichment with ALA alone or supplemented with *B. breve* NCIMB 702258 and *B. breve* DPC 6330 on tissue fatty acid composition

To investigate the effects of the ALA enriched diet on tissue fatty acid composition, fatty acid profiling (saturated and unsaturated) was performed on liver and epididymal adipose tissue. Following 6 weeks of dietary intervention, all ALA enriched groups had increased caprylic acid (C8:0) in the liver (*P* < 0.05; Table 2), compared with CON. For unsaturated fatty acids, all ALA enriched groups had increased stearidonic acid (C18:4) in the liver (*P* < 0.05; Table 2), increased ALA (C18:3*n*-3) and EPA (C20:5) in both liver (*P* < 0.05; Table 2) and adipose tissue (*P* < 0.05; Table 3) and decreased arachidonic acid (C20:4) and oleic acid (C18:1) in adipose tissue (*P* < 0.05; Table 3), compared with CON.

Comparing the effects of dietary supplementation with ALA + NCIMB 702258 with ALA-CON, there were significant increases in the saturated fatty acids caprylic acid (*P* < 0.05; Table 2), capric acid (C10:0; *P* < 0.05; Table 2), lauric acid (C12:0; *P* < 0.05; Table 2) and myristic acid (C14:0; *P* < 0.05; Table 2) in liver and increased pentadecyclic acid (C15:0; *P* < 0.05; Table 3) and stearic acid (C18:0; *P* < 0.05; Table 3) in adipose tissue. Oleic acid was decreased in adipose tissue (*P* < 0.05; Table 3), while the PUFAs EPA (*P* < 0.05; Table 3) and DHA (C22:6; *P* < 0.05; Table 3) were increased and DHA was increased in liver (*P* < 0.05; Table 2) in ALA + NCIMB 702258, compared with ALA-CON.

Supplementation of the ALA enriched diet with *B. breve* DPC 6330 decreased stearic acid in liver (*P* < 0.05; Table 2) and increased pentadecyclic acid in adipose tissue (*P* < 0.05; Table 3), compared with ALA-CON. *B. breve* DPC 6330 also increased hepatic linoleic acid (C18:2; *P* < 0.05; Table 2) and DHA in the liver (*P* < 0.05; Table 2) and adipose tissue (*P* < 0.05; Table 3) and decreased adipose tissue oleic acid (*P* < 0.05; Table 3) compared with ALA-CON.

Unexpectedly, the *n*-3 PUFA score was significantly increased in all ALA enriched groups compared with CON, irrespective of microbial supplementation (*P* < 0.05; Table 2). It was found that hepatic peroxisomal beta oxidation was significantly increased following supplementation of the ALA enriched diet with *B. breve* (both strains), compared with CON and ALA-CON (*P* < 0.05; Table 2). This result correlated with significantly reduced hepatic levels of CNLA (*P* < 0.05) and *c*9*t*11CLA (*P* < 0.05) following supplementation with *B. breve* compared with CON and ALA-CON (Table 2). Interestingly, CNLA (*P* < 0.05) and *c*9*t*11CLA (*P* < 0.05) were increased in adipose tissue following supplementation with both *B. breve* strains compared with CON and ALA-CON (Table 3).

### Strain specific effects of *B. breve* on endocannabinoid and congener levels in liver and adipose tissue

**Figure 1:**
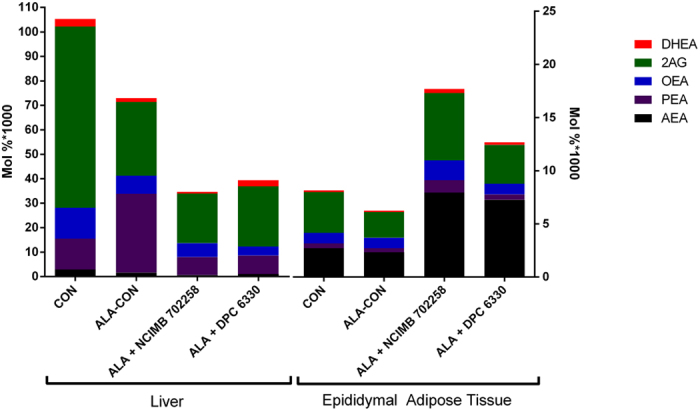
Endocannabinoid levels (%mol) in the liver and epididymal adipose tissue of mice fed an ALA enriched diet either alone or in combination with *Bifidobacterium breve* NCIMB 702258 or *B. breve* DPC 6330 or an unsupplemented diet for 6 weeks. Actual values and significant differences are highlighted in Supplementary Table 1. AEA, N-arachidonoylethanolamide; PEA, N-palmitoylethanolamide; OEA, oleoylethanolamide; 2AG, 2-arachidonoylglycerol; DHEA, docosahexaenoylethanolamide.

To understand the dietary and microbial impact of ALA and *B. breve* on the ECS, liver and adipose tissue levels of endocannabinoids (EC) and their congeners were determined. Hepatic levels of AEA, oleoylethanolamide (OEA) and 2-AG were reduced in all ALA enriched dietary groups, compared with CON (*P* < 0.05; Supplementary Table 1 (displaying significance) and Fig. 1). Furthermore, both *B. breve* strains reduced AEA and N-palmitoylethanolamide (PEA) levels in liver, compared with ALA-CON (*P* < 0.05; Supplementary Table 1 and Fig. 1), while *B. breve* DPC 6330 also reduced hepatic OEA levels, compared with ALA-CON (*P* < 0.05; Supplementary Table 1 and Fig. 1).

*B. breve* supplementation had a profound effect on EC and congener levels in adipose tissue. Both strains increased AEA and docosahexaenoylethanolamide (DHEA) levels here compared with CON and ALA-CON (*P* < 0.05; Supplementary Table 1 and Fig. 1). Interestingly, a greater effect of *B. breve* NCIMB 702258 on adipose tissue EC levels was identified, as this strain also increased PEA (*P* < 0.05) and OEA (*P* < 0.05) levels compared with CON and ALA-CON (Supplementary Table 1 and Fig. 1).

### Impact on mRNA gene expression levels of enzymes involved in *n*-3 PUFA metabolism within liver and uptake of fatty acids to liver following dietary enrichment with ALA either alone or supplemented with *B. breve* NCIMB 702258 or *B. breve* DPC 6330

**Figure 2:**
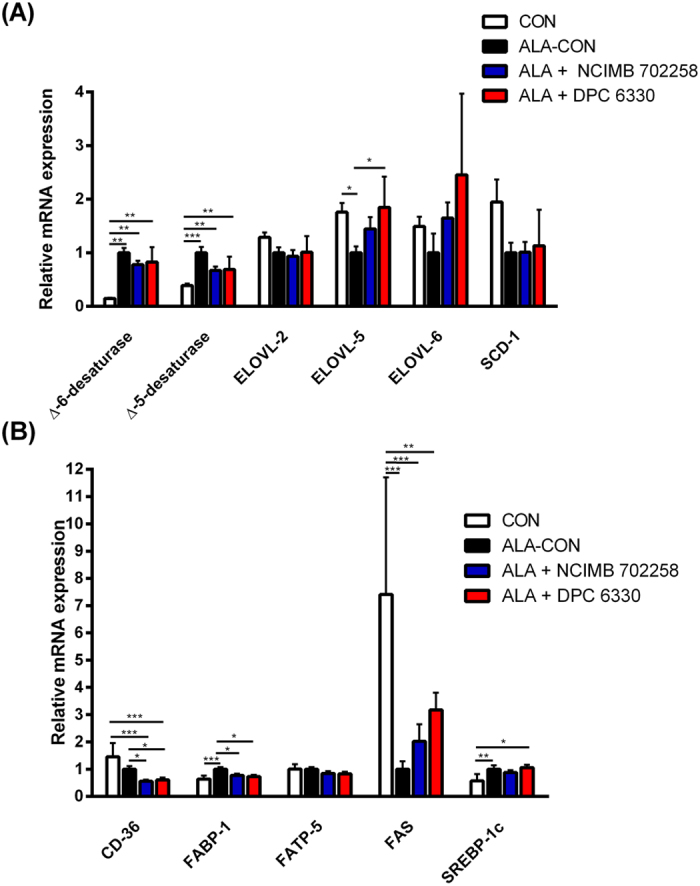
(A) Relative mRNA gene expression of fatty acid metabolism enzymes and (B) fatty acid uptake enzymes, in the livers of mice fed ALA enriched diets either alone (*n* = *10*) or in combination with *Bifidobacterium breve* NCIMB 702258 (*n* = *10*) or *B. breve* DPC 6330 (*n* = *10*), or an unsupplemented diet (*n* = *10*), relative to β-actin. Expression in the unsupplemented and ALA enriched diets supplemented with either *B. breve* NCIMB 702258 or *B. breve* DPC 6330 is relative to the ALA enriched diet, which was set to 1. Values are means ± SD, represented by vertical bars. Data was analysed by one-way ANOVA, followed by *post hoc* Tukey’s multiple comparison test. Significant differences are represented by *(*P* < 0.05), **(*P* < 0.05), ***(*P* < 0.05).

The mRNA expression of enzymes involved in *n*-3 PUFA metabolism within liver (Fig. 2(A)) and genes involved in the transport and uptake of fatty acids to the liver (Fig. 2(B)) were determined using real-time PCR. An increase in hepatic mRNA expression of Δ-6-desaturase was found in all ALA enriched groups, compared with CON (*P* < 0.01; Fig. 2(A)). Hepatic mRNA expression of elongation of very long chain fatty acids (ELOVL)-5 was reduced in ALA-CON compared with CON (*P* < 0.05; Fig. 2(A)). Hepatic mRNA expression of sterol regulatory element binding protein (SREBP)-1c was increased following supplementation with ALA, compared with CON (*P* < 0.05; Fig. 2(B)). The CON group exhibited increased hepatic mRNA expression of fatty acid synthase (FAS), compared with all ALA enriched groups (*P* < 0.01; Fig. 2(B)).

Hepatic mRNA expression of ELOVL-5 was elevated in ALA + DPC 6330 (*P* < 0.05; Fig. 2(A)), compared with ALA-CON. ALA + NCIMB 702258, ALA + DPC 6330 and CON demonstrated significantly reduced hepatic mRNA expression of Δ-5-desaturase, compared with ALA-CON (*P* < 0.01; Fig. 2(A)).

Both ALA + NCIMB 702258 and ALA + DPC 6330 exhibited decreased hepatic mRNA expression of CD-36, compared with ALA-CON and CON (*P* < 0.05; Fig. 2(B)). In addition, ALA + NCIMB 702258 and ALA + DPC 6330 had decreased hepatic mRNA expression of fatty acid binding protein (FABP)-1, compared with ALA-CON (*P* < 0.05; Fig. 2(B)). Hepatic mRNA expression of SREBP-1c was increased in ALA + DPC 6330, compared with CON (*P* < 0.05; Fig. 2(B)).

### Impact on mRNA gene expression levels of enzymes involved in the transport and uptake of fatty acids to the ileum following dietary enrichment with ALA either alone or supplemented with *B. breve* NCIMB 702258 or *B. breve* DPC 6330

**Figure 3:**
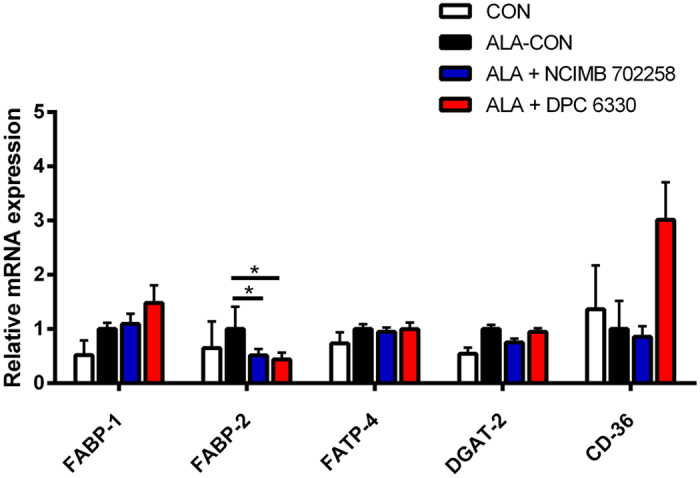
Relative mRNA gene expression of fatty acid uptake and transport enzymes in the ileum of mice fed ALA enriched diets either alone (*n* = *10*) or in combination with *Bifidobacterium breve* NCIMB 702258 (*n* = *10*) or *B. breve* DPC 6330 (*n* = *10*), or an unsupplemented diet (*n* = *10*), relative to β-actin. Expression in the unsupplemented diet and ALA enriched diets supplemented with either *B. breve* NCIMB 702258 or *B. breve* DPC 6330 is relative to the ALA enriched diet, which was set to 1. Values are means ± SD, represented by vertical bars. Data was analysed by one-way ANOVA, followed by *post hoc* Tukey’s multiple comparison test. Significant differences are represented by *(*P* < 0.05).

ALA + NCIMB 702258 and ALA + DPC 6330 decreased ileal mRNA gene expression of FABP-2, compared with ALA-CON (*P *< 0.05; Fig. 3). Although not significant, the expression of FABP-1 and CD-36 were increased in ALA + DPC 6330 (Fig. 3). The expression of fatty acid transport protein (FATP)-4 and diacylglycerol O-acyltransferase (DGAT)-2 were unchanged between all groups (Fig. 3).

## Discussion

This study investigated the impact of dietary ALA enrichment with or without fatty acid conjugating *B. breve* supplementation on fat composition/distribution and the mechanisms through which commensal gut microbes alter lipid metabolism. The work presented herein tested the hypothesis that the alterations in host tissue fatty acid composition by *B. breve* could be linked to modulation of fat absorption processes in the small intestine and/or metabolism of fatty acids to longer-chain unsaturated derivatives in the liver.

Previously, we reported that dietary supplementation with linoleic acid and *B. breve* NCIMB 702258 significantly increased bioactive *c*9*t*11CLA concentrations in the liver of mice^16^, while dietary supplementation with *B. breve* NCIMB 702258 together with ALA increased tissue *n*-3 LC-PUFA in mice, compared with those that did not receive the strain^17^. More recently, we demonstrated an increase in brain DHA associated with *B. breve* NCIMB 702258 supplementation in mice^18^.

In general, ALA is a poor precursor for *n*-3 LC-PUFA, particularly DHA, first due to the efficiency for ALA to undergo beta-oxidation in the mitochondria^40^, rendering this fatty acid less available for desaturation and elongation and second because biosynthesis of DHA requires a crucial step in the peroxisome for partial beta-oxidation^40^. Therefore, any other event that increases desaturation and peroxisomal beta-oxidation would also favour DHA biosynthesis. Our recent data demonstrate that dietary CLA, in a specific range of ratio with ALA, significantly increased DHA biosynthesis (unpublished data), rendering some food, for example CLA enriched cheese, an unexpected source of DHA. Furthermore, associations between a reduced *n*-3 PUFA score following consumption of a CLA enriched cheese, decreased plasma AEA and an improved blood lipid profile in hypercholesterolaemic patients have been described^44^, thus exposing a link between *n*-3 PUFA, CLA, the ECS and adiposity. Interestingly, dietary CLA in a model of fatty liver was associated with increased hepatic PEA and OEA^45^. Based on these data, we were prompted to evaluate whether *B. breve*, possibly by increasing CLA formation, could modify the tissue fatty acid profile (as before), but this time identify whether EC and their congener biosynthesis were also affected. While CLA was increased in adipose tissue following supplementation of the ALA enriched diet with both *B. breve* strains, no change to the CLA concentration was observed in the liver, most likely due to increased hepatic peroxisomal beta-oxidation to CD16:2. This was demonstrated by the increased ratio of CD16:2: *c*9*t*11CLA, however we did not detect a subsequent increase in hepatic DHA concentration. Furthermore, we demonstrated that dietary ALA supplementation increased liver and adipose tissue ALA and EPA with decreased arachidonic acid levels in the adipose tissue. Highly polyunsaturated *n*-3 fatty acids replace arachidonic acid as an eicosanoid substrate in cell membranes^46^, resulting in decreased production of arachidonic acid-derived proinflammatory eicosanoids such as prostaglandin E_2_ and leukotriene B_4_^47^ thus, increased tissue EPA could be beneficial in a variety of chronic inflammatory settings such as non-alcoholic fatty liver disease (NAFLD) and IBD. The overall hepatic *n*-3 PUFA score was unsurprisingly increased in all ALA enriched dietary groups, compared with the control. *B. breve* NCIMB 702258 in combination with the ALA enriched diet increased EPA and DHA in adipose tissue, while *B. breve* DPC 6330 increased DHA only in the adipose tissue, compared with the ALA enriched control group.

Analysis of the EC revealed a decrease in hepatic AEA, OEA and 2-AG in all ALA enriched groups, compared with the control. Furthermore, both *B. breve* strains further reduced AEA and PEA in the liver, compared with the ALA enriched control group. In contrast, further supplementation of the ALA enriched diet with *B. breve* NCIMB 702258 specifically increased AEA, PEA, OEA and DHEA in the adipose tissue. These data suggest that the effect of *B. breve* NCIMB 702258 on the ECS may be similar to those triggered by inflammation. From our experimental conditions, intake of *B. breve* NCIMB 702258 triggered a cascade linked to activation of toll-like receptors (TLRs)^48^, but we cannot rule out the possibility that some of the effects might be mediated by CLA, such as the increase in adipose tissue PEA and OEA, as previously demonstrated in the liver of rats fed CLA^45^. Interestingly, feeding of both strains increased CLA in adipose tissue. However, only *B. breve* NCIMB 702258 enhanced AEA, PEA, OEA and DHEA adipose tissue levels, which suggest that even among strains of the same species, different metabolic responses occur on the ECS. The increased adipose tissue AEA could be related to TLR activation as previously demonstrated in lymphocytes, where fatty acid amide hydrolase activity was downregulated^49^. Therefore, these data suggest that concurrent effects of these nutritional factors may result in changes to the ECS.

We have demonstrated an ALA enriched dietary effect on the hepatic mRNA expression of Δ-6-desaturase. This enzyme is involved in the biosynthetic pathway of long-chain, highly unsaturated PUFA^50^ and so may partly explain the increased EPA concentrations in the liver and adipose tissue. Beyond docosapentaenoic acid formation, Δ-6-desaturase also metabolises tetracosapentaenoic acid (C24:5*n*-3), a precursor to DHA, however since Δ-6-desaturase is used by two substrates in the same pathway of *n*-3 PUFA metabolism, there is intrinsic potential for competitive substrate inhibition to occur^51^. Previous studies revealed that high dietary ALA content up-regulates hepatic Δ-6-desaturase gene expression levels in different models^42^,^52^. The hepatic mRNA expression of ELOVL-5 was not found to be increased for all ALA enriched dietary groups. One recent study demonstrating the effect of ALA supplementation on Δ-6-desaturase and ELOVL-5 expression in the common carp showed that after 42 days, while Δ-6-desaturase activity decreased, there was an increase in ELOVL-5 expression^42^. This suggests that while the activity of one rate-limiting enzyme involved in the metabolism of *n*-3 PUFA increases, there may not be a corresponding increase in the activity of the other at the same time, thus the sequence of enzyme activity is time dependent.

Supplementation of *B. breve* DPC 6330 in an ALA enriched diet had a significant effect on increasing the mRNA expression of genes involved in the transport and uptake of fatty acids to the distal small intestine. The mRNA expression of FABP-1 and CD-36 were increased in the ileum following supplementation of the ALA enriched diet with *B. breve* DPC 6330. FABP-1 and CD-36 glycoprotein, which play a role in cellular adhesion and in the regulation of fatty acid transport and uptake, have previously been shown to be increased in the intestine following *in vivo* exposure of human intestinal mucosa to the probiotic species *Lactobacillus plantarum* WCFSI^53^. Furthermore, the role of the microbiota in stimulating dietary fatty acid absorption in the intestinal epithelium have previously been demonstrated^54^.

Hepatic TAG levels were reduced following 6 weeks of dietary intervention with the ALA enriched diet. It has been reported that EPA may be a promising novel therapy to decrease hepatic TAG^55^,^56^ and may therefore be of importance for the treatment of NAFLD. Coincidently, all ALA dietary groups were associated with increased hepatic EPA. In addition, all ALA enriched dietary groups had reduced hepatic FAS mRNA expression and lower SCD-1 expression whereby the decrease in SREBP-1c regulated enzymes had a significant effect on fatty acid synthesis in the liver^57^. Supplementation of the ALA enriched diet with both *B. breve* strains decreased hepatic TAG levels compared with the ALA only group. These data demonstrate a potential for administered *B. breve* to reduce liver TAG levels in the host. Hepatic TAG levels, the mRNA expression of CD-36 and FABP-1 were all decreased following further supplementation of the ALA enriched diet with *B. breve*, compared to the ALA enriched group, whereby CD-36 and FABP-1 are involved in transport and uptake of fatty acids to the liver^58^,^59^. This indicates a strain-specific effect of these two *B. breve* strains toward reducing hepatic TAG levels while also reducing the hepatic mRNA expression of genes relating to reduced fatty acid uptake to the liver. A potential therapeutic role for commensal bacteria in the treatment of NAFLD have been described^60^. For example, it was reported that dietary supplementation with *Lactobacillus plantarum* MA2 with a cholesterol enriched diet resulted in reduced liver TAG and increased numbers of fecal *Lactobacillus* and *Bifidobacterium* in rats^61^. Furthermore, the potential of VSL#3, a multistrain preparation composed of *Streptococcus thermophilus* and several strains of *Lactobacillus* and *Bifidobacterium* for reducing inflammation (reduced TNFα and cyclooxygenase expression, increased PPARα expression) and oxidative damage (inducible nitric oxide synthase) to the liver while ameliorating the liver lipid profiles has been shown^62^.

The data presented in this study confirmed that dietary ALA positively impacted on tissue fatty acid composition, increased hepatic *n*-3 PUFA metabolism and reduced hepatic TAG levels, hepatic fatty acid synthesis enzymes and hepatic uptake of fatty acids and demonstrated effects on the ECS. Previously, we demonstrated that *B. breve* NCIMB 702258 in an ALA enriched diet increased hepatic EPA concentrations, compared with ALA alone^17^. We have elaborated on these previous findings by demonstrating an impact not only of *B. breve* NCIMB 702258 in an ALA enriched diet, but also of *B. breve* DPC 6330 toward reducing liver triglyceride levels, altering fatty acid and EC profiles and expression of key enzymes involved in the metabolism of fatty acids. Supplementation with either *B. breve* strain in combination with an ALA enriched diet reduced liver TAG levels which was associated with reduced hepatic CD-36 and FABP-1 mRNA expression, involved in the uptake of fatty acids to the liver. While it is clear from the data that ALA impacted on desaturase activity, there was little effect of *B. breve* supplementation on *n*-3 PUFA metabolism gene expression in the liver. Instead, the increased peroxisomal beta-oxidation caused by the strains, could explain why there was no increase in CLA identified in the liver. Adipose tissue CLA was increased, as a result of *B. breve* supplementation. Future studies will be devoted to evaluating the possible synergistic effects of CLA enriched products with *B. breve*: (i) in maintaining/re-equilibrating energy and lipid metabolism, by modulating tissue EC and congener profiles, (ii) acting on energy metabolism and EPA and DHA related molecules, and (iii) to assess if there is an optimal level of dietary EPA and DHA to allow such a synergistic activity. This novel study identifies mechanisms through which these lipid-modulating *Bifidobacterium* strains may be acting *in vivo*. Considering that the relative proportions of *Bifidobacterium* in the gut microbiota of C57BL/6 J have previously been reported to compose 2–3% only of the total gut microbial content^18^ (mainly *Bifidobacterium animalis* and *Bifidobacterium pseudolongum*^63^), it is indeed intriguing to identify probiotic manipulations of the gut microbiota which can have a positive impact on health. This nutritional approach may prove helpful in the alleviation of metabolic disorders such as those causing NAFLD, where CLA^45^, *n*-3 PUFA^64^,^65^ and manipulation of the gut microbiota^66^ have been shown to decrease adiposity accumulation in the liver.

## Methods

### Preparation and administration of *B. breve* NCIMB 702258 and *B. breve* DPC 6330

Rifampicin resistant variants of *B. breve* NCIMB 702258 and *B. breve* DPC 6330 were isolated by spread-plating ~10^9^ colony forming units (CFU) from an overnight culture onto MRS agar (de Man, Rogosa & Sharpe; Difco Laboratories, Detroit, MI, USA), supplemented with 0.05% (w/v) L-cysteine hydrochloride (Sigma-Aldrich, Wicklow, Ireland) (mMRS), containing 500 μg/mL rifampicin (Sigma-Aldrich). Following anaerobic incubation (anaerobic jars with Anaerocult A gas packs; Merck, Darmstadt, Germany) at 37 °C for 3 days, colonies were stocked in mMRS broth containing 40% (v/v) glycerol and stored at −80 °C. To confirm that the rifampicin resistant variants were identical to the parent strain, molecular fingerprinting using pulse-field gel electrophoresis was employed.

Prior to freeze drying, *B. breve* NCIMB 702258 and *B. breve* DPC 6330 were grown in mMRS by incubating for 48 hr at 37 °C under anaerobic conditions. The culture was washed twice in phosphate buffered saline (PBS) and resuspended at a concentration of ~2 × 10^10^ cells/mL in 15% (w/v) trehalose (Sigma-Aldrich) in dH_2_O. One millilitre aliquots were freeze-dried using a 24 hr programme (freeze temp. −40 °C, condenser set point −60, vacuum set point 600 m Torr). Animals were each administered approximately 1 × 10^9^ live microorganisms per day, which was achieved by resuspending appropriate quantities of freeze-dried powder in water which was consumed *ad libitum*. Mice that did not receive the bacterial strains received placebo freeze-dried powder (15% (w/v) trehalose in dH_2_O). Water containing either the bacterial strains or placebo freeze-dried powder was the only water supplied to the mice throughout the trial. Freeze dried powders with the bacterial strains underwent continuous quality control checks of cell counts for the duration of the trial, by plating serial dilutions on mMRS agar supplemented with 100 μg/mL of mupirocin (Oxoid) and 100 μg/mL rifampicin (Sigma-Aldrich) and incubating plates anaerobically for 72 hr at 37 °C.

### Animals and Treatment

All animal experiments were approved by University College Cork (UCC) Animal Ethics Committee and experimental procedures were conducted under the appropriate license from the Irish Government. Female BALB/c mice were purchased from Harlan Ltd. (Briester, Oxon, UK) at 8 weeks of age and housed under barrier-maintained conditions within the Biological Services Unit, UCC. Mice were allowed to acclimatise for 1 week prior to commencement of the study and were fed *ad libitum* with Teklad Global Rodent Standard Diet (Harlan Laboratories, Madison, WI, USA, #2018 S), with free access to water at all times. Mice were housed in groups of five per cage and maintained in a controlled environment at 25 °C under 12-hr-light/12-hr-dark cycle. After 1 week of acclimatisation, animals were randomly divided into four groups (*n* 10 mice per group): (1) unsupplemented control group (**CON**) fed a standard rodent diet with placebo freeze-dried powder (15% w/v trehalose in drinking water); (2) flaxseed oil (FXO, enriched in ALA) supplemented group (**ALA-CON**) with placebo freeze-dried powder (15% w/v trehalose in drinking water); (3) FXO supplemented group with *B. breve* NCIMB 702258 (**ALA + NCIMB 702258**) (approximate daily dose of 10^9^ microorganisms); (4) FXO supplemented group with *B. breve* DPC 6330 (**ALA + DPC 6330**) (approximate daily dose of 10^9^ microorganisms).

The unsupplemented control diet contained the following composition: corn starch (32.45%), casein (20.0%), sucrose (15.0%), maltodextrin (12.0%), cellulose (5.0%), mineral mix (3.5%), vitamin mix (1.5%), L-cysteine (0.3%), choline bitartrate (0.25%), TBHQ antioxidant (0.002%) and the following composition of fat: palm oil (3.0%), safflower oil (3.0%), olive oil (3.0%), FXO (1.0%). The *n*-6:*n*-3 ratio of this diet was ~5.3 and the diet contained ~0.5% ALA. The FXO supplemented diet contained the following nutrient composition: corn starch (32.45%), casein (20.0%), sucrose (15.0%), maltodextrin (12.0%), cellulose (5.0%), mineral mix (3.5%), vitamin mix (1.5%), L-cysteine (0.3%), choline bitartrate (0.25%), TBHQ antioxidant (0.002%) and the following composition of fat: FXO (5.5%), palm oil (1.5%), safflower oil (1.5%), olive oil (1.5%). The *n*-6:*n*-3 ratio of this diet was ~0.75 and the diet contained ~3% ALA.

Body weight and food intake were assessed weekly. Following 6 weeks on experimental diets, the animals were sacrificed by cervical dislocation. Liver, brain, fat pads (epididymal, perirenal and mesenteric), gastrointestinal tract from stomach to anus, and cecal contents were removed, blotted dry on filter paper, weighed and flash-frozen immediately in liquid nitrogen. All samples were stored at −80 °C until processed. Blood samples were collected into serum collection tubes (BD Diagnostics, Oxford, UK) from fasted animals, and allowed to clot for at least 30 min at 4 °C before centrifugation for 20 min at 10,000 *g* to separate the serum.

### Culture Dependent Microbial Analysis

Fresh fecal samples were taken from BALB/c mice every second week for microbial analysis. Microbial analysis of the fecal samples involved enumeration of the *B. breve* strains by plating serial dilutions on MRS agar supplemented with 100 μg/mL of mupirocin (Oxoid), 100 μg/mL of rifampicin (Sigma-Aldrich) and 50 units/mL of nystatin (Sigma-Aldrich). Agar plates were incubated anaerobically for 72 h at 37 °C.

### Lipid Extraction and Fatty Acid Analysis

An aliquot of the lipid fraction for each sample was mildly saponified using a procedure in order to obtain free fatty acids (FFA) for high performance liquid chromatography (HPLC) analysis. Lipid extracts were dissolved in 5 ml of ethanol, 100 μl of desferal (25 mg/ml ddH_2_O), 1 ml of a 25% (v/v) solution of ascorbic acid in water, 0.5 ml of 10 N KOH, and left for 14 h in the dark at room temperature. Following this, 10 ml of *n*-hexane and 7 ml of ddH_2_O were added. The samples were then acidified with 0.35 ml of 37% (v/v) HCI, to pH 3–4. Samples were centrifuged for 1 h at 900 *g*. The hexane phase containing FFAs was collected, the solvent evaporated, and the residue was dissolved in 0.5 ml of CH_3_CN/0.14% (v/v) CH_3_COOH.

Separation of FAs, including CLA and its metabolites, was performed on an Agilent 1100 HPLC system (Agilent, Palo Alto, Calif., USA) equipped with a diode array detector. A C-18 Inertsil 5 ODS-2 Chrompack column (Chrompack International BV, Middleburg, The Netherlands), 5 μm particle size, 150 × 4.6 mm, was used with a mobile phase of CH_3_CN/H_2_O/CH_3_COOH (70/30/0.12, v/v/v) at a flow rate of 1.5 ml/min^67^. Conjugated diene unsaturated FAs were detected at 234 nm. Spectra (195–315 nm) of the eluate were obtained every 1.28 s and were electronically stored. Second-derivate UV spectra of the conjugated diene FAs were generated using Phoenix 3D HP Chemstation software (Agilent, Palo Alto, CA). These spectra were acquired to confirm identification of the HPLC peaks^68^.

Since SFAs are transparent to UV, after derivatization, they were measured as fatty acid methyl esters (FAME) using gas chromatography (Agilent, Model 6890, Palo Alto, CA) equipped with split ratio of 20:1 injection port, a flame ionization detector (FID), an autosampler (Agilent, Model 7673), a 100 m HP-88 fused capillary column (Agilent). Finally, data were analysed by the Agilent ChemStation software system. The injector and detector temperatures were set up at 250 °C and 280 °C, respectively. H_2_ served as carrier gas (1 ml/min), and the FID gases were H_2_ (30 ml/min), N_2_ (30 ml/min), and purified air (300 ml/min). The temperature program was as follows: initial temperature was 120 °C, programmed at 10 °C/min to 210 °C and 5 °C/min to 230 °C, then programmed at 25 °C/min to 250 °C and held for 2 min^64^.

### Measurement of TAG in the Liver and Serum

The lipids from 50 mg of frozen liver were extracted and purified according to the method of Folch^69^. Liver lipids were extracted using chloroform-methanol (2:1, v:v; Thermo Scientific) and an aliquot of the organic phase was collected, dried and resuspended in duplicate in Infinity-TAG lipid-stable reagent (Thermo Scientific). TAG levels were determined according to the manufacturer’s instructions and lipids were quantified using a TAG chemistry calibrator (Pointe Scientific Inc., MI, USA). Serum TAG levels were analysed in duplicate using the commercial L-Type TAG M kit (Wako Diagnostics, Neuss, Germany).

### Analysis of Endocannabinoids and their Congeners

Aliquots of the organic phase containing extracted lipids were evaporated to dryness under vacuum, and reconstituted with 0.4 ml of 100% (v/v) methanol. Quantification of AEA, 2-AG, PEA and OEA, was performed by liquid chromatography-atmospheric pressure chemical ionization-mass spectrometry (LC-APCI-MS), and using selected ion monitoring (SIM) at M + 1 values for the four compounds and their deuterated homologues^70^. A C-18 Zorbax Eclipse Plus column (Agilent, Palo Alto, CA) 5 μm particle size, 50 × 4.6 mm was used with a mobile phase of CH_3_OH/H_2_O/CH_3_COOH (80/20/0.3, v/v/v) at a flow rate of 0.5 ml/min.

### RNA Extractions and Complementary DNA Synthesis

Total RNA was isolated from liver and ileum using the commercial RNeasy Mini-Kit (Qiagen, West Sussex, UK), according to the manufacturer’s instructions. Total isolated RNA was quantified using the Nanodrop (Thermo Scientific). Single stranded complementary DNA (cDNA) was synthesised from 1 μg of total RNA using 2.5 ng/μL random primers (Promega, WI, USA), 10 mM PCR nucleotide mix (Promega), 40 units/μL RNasin Plus RNase inhibitor (Promega) and the Im-Prom II reverse transcriptase (Promega).

### Real-Time PCR Analysis

Amplification of generated cDNA was performed in the Lightcycler 480 system (Roche Diagnostics Ltd., West Sussex, UK) using 0.25 μM primers (MWG Eurofins, Ebersberg, Germany), 1 μL cDNA and the Lightcycler 480 SYBR Green I Master kit (Roche Diagnostics Ltd). Real-time PCR conditions were set at: 95 °C for 10 min followed by 50 cycles at 95 °C for 10 sec, 60 °C for 5 sec and 72 °C for 15 sec. Specific forward and reverse primers used to amplify cDNA were newly designed and are listed in Table 4. All samples were analysed in duplicate and normalised to β-actin, as a constitutively expressed control gene. Melting curve analysis allowed the validation of the authenticity of the real-time PCR products. Basic relative quantification of expression was determined using the comparative 2^−ΔΔCt^ method.

### Statistical Analysis

All results are presented as means ± SD (per group). To assess whether differences between the treatment groups were significant, data were analysed using one-way ANOVA followed by *post hoc* Tukey’s multiple comparison test using GraphPad Prism version 4.0 for Windows (GraphPad Software). Statistical significance was accepted at *P* < 0.05. Grubbs method was used to test for outliers^71^.

## Additional Information

**How to cite this article**: Patterson, E. *et al. Bifidobacterium breve* with α-linolenic acid alters the composition, distribution and transcription factor activity associated with metabolism and absorption of fat. *Sci. Rep.* **7**, 43300; doi: 10.1038/srep43300 (2017).

**Publisher's note:** Springer Nature remains neutral with regard to jurisdictional claims in published maps and institutional affiliations.

## Supplementary Material

SUPPLEMENTARY TABLE 1

## Figures and Tables

**Table 1:** Body mass, fat mass, liver mass, liver and serum triglyceride (TAG) levels in mice fed an ALA enriched diet either alone or in combination with *Bifidobacterium breve* NCIMB 702258 or *B. breve* DPC 6330 or an unsupplemented diet for 6 weeks.

	CON		ALA-CON		ALA+NCIMB 702258		ALA+DPC 6330	
	Mean	SD	Mean	SD	Mean	SD	Mean	SD
Initial weight (g) *n* = *10*	19.06^a,b^	0.39	19.82^a^	0.49	19.05^b^	0.90	19.52^a,b^	0.63
Final weight (g) *n* = *10*	20.37	0.72	21.13	0.66	20.21	1.10	21.19	0.96
Weight gain (%) *n* = *10*	6.87	3.55	6.65	3.25	6.12	3.36	8.54	3.32
Visceral fat mass (g)^2^ *n* = *9*	0.61	0.16	0.75	0.14	0.70	0.17	0.78	0.21
Liver TAG (mg/g) *n* = *10*	16.26^b^	2.49	12.89^a^	1.71	10.03^c^	2.03	9.79^c^	1.12
Serum TAG (mg/dL) *n* = *10*	80.21	31.68	58.31	16.38	73.62	14.45	70.98	16.60

^a,b,c^Mean values within a row with unlike superscript letters were significantly different (*P* < 0.05 ANOVA followed by *post hoc* Tukey’s multiple comparison test). ^2^Includes epididymal, perirenal and mesenteric fat pads.

**Table 2:** Fatty acid profile (% mol) in the liver of mice fed an ALA enriched diet either alone or in combination with *Bifidobacterium breve* NCIMB 702258 or *B. breve* DPC 6330 or an unsupplemented diet for 6 weeks.

Fatty acid	CON		ALA-CON		ALA +NCIMB 702258		ALA +DPC 6330	
	*n* = *9*		*n* = *9*		*n* = *8*		*n* = *9*	
	*mol %*		*mol %*		*mol %*		*mol %*	
SFA	Mean	SD	Mean	SD	Mean	SD	Mean	SD
8:0	1.95^a^	0.53	2.78^b^	0.89	3.82^c^	0.74	2.27^b,c^	1.01
10:0	0.63^a^	0.21	0.75^a^	0.23	1.49^b^	0.40	0.91^a^	0.49
12:0	0.40^a^	0.17	0.47^a^	0.12	0.81^b^	0.16	0.47^a^	0.16
14:0	0.65^a^	0.17	0.65^a^	0.20	1.07^b^	0.23	0.65^a^	0.25
15:0	0.42	0.24	0.41	0.14	0.61	0.32	0.54	0.28
16:0	29.10	5.44	25.98	5.64	28.54	1.90	28.82	1.10
18:0	12.43^a,b^	2.06	14.43^a^	4.06	11.94^a,b^	0.89	10.96^b^	1.65
Total SFA	45.58	4.36	45.47	2.63	48.29	3.71	44.62	2.28
PUFA								
16:1*t*	5.32	1.01	6.37	3.62	ND	ND	ND	ND
18:1	19.87	2.93	19.25	5.45	16.14	1.02	16.43	1.92
18:2	13.19^a,b^	1.67	11.83^a^	2.44	13.83^a,b^	1.66	15.10^b^	0.93
18:3*n*-3	0.46^a^	0.06	3.62^b^	0.43	3.33^b^	0.36	3.54^b^	0.35
18:3*n*-6	0.27	0.05	ND	ND	ND	ND	ND	ND
18:4	0.04^a^	0.01	0.13^b^	0.02	0.14^b^	0.03	0.13^b^	0.02
20:3*n*-6	0.82	0.13	0.94	0.22	1.18	0.16	1.32	0.15
20:3*n*-9	0.07	0.02	0.05	0.01	0.07	0.02	0.06	0.01
20:4	10.18^a^	1.30	5.78^b^	1.55	6.12^a,b^	0.99	6.90^a,b^	0.96
20:5	0.29^a^	0.09	1.95^b^	0.52	2.63^a^	0.40	2.92^a^	0.46
22:5	0.05	0.01	ND	ND	ND	ND	ND	ND
22:5*n*-3	0.75	0.47	1.07	0.27	1.30	0.25	1.40	0.47
22:4	0.14^a^	0.03	0.05^b^	0.01	0.06^a,b^	0.03	0.06^a,b^	0.01
22:6	7.64^a^	0.83	6.45^b^	1.39	8.05^a^	1.02	8.79^a^	0.96
*n*-3 PUFA score	41.47^a^	1.38	55.46^b^	5.43	59.05^b^	2.26	58.46^b^	2.22
Peroxisomal β-oxidation	93.96^a^	18.09	84.22^a^	12.40	153.75^b^	52.47	226.18^b^	159.85
Total hydroperoxides	0.07	0.02	0.10	0.08	0.05	0.02	0.05	0.01
CD16:3	0.005	0.004	0.010	0.010	0.010	0.003	0.010	0.010
CD16:2	0.04	0.01	0.04	0.01	0.04	0.02	0.04	0.01
CD 18:3 (CNLA)	0.06^a^	0.02	0.06^a^	0.02	0.03^b^	0.01	0.02^b^	0.004
CLA 9,11	0.04^a^	0.01	0.04^a^	0.01	0.03^b^	0.02	0.03^b^	0.01

SFA, saturated fatty acid; PUFA, polyunsaturated fatty acid; CD, conjugated diene; CNLA, conjugated α-linolenic acid; CLA, conjugated linoleic acid; ND, not detected. ^a,b,c^Mean values within a row with unlike superscript letters were significantly different (*P* < 0.05 ANOVA followed by *post hoc* Tukey’s multiple comparison test).

**Table 3:** Fatty acid profile (% mol) in the epididymal adipose tissue of mice fed an ALA enriched diet either alone or in combination with *Bifidobacterium breve* NCIMB 702258 or *B. breve* DPC 6330 or an unsupplemented diet for 6 weeks.

Fatty acid	CON		ALA-CON		ALA+NCIMB 702258		ALA+DPC 6330	
	*n* = *9*		*n* = *9*		*n* = *8*		*n* = *9*	
	*mol%*		*mol%*		*mol%*		*mol%*	
SFA	Mean	SD	Mean	SD	Mean	SD	Mean	SD
8:0	3.03	0.54	3.55	1.90	6.55	8.34	3.36	0.54
10:0	0.72	0.19	1.26	0.77	2.13	2.61	1.74	0.95
12:0	0.69	0.25	0.86	0.30	1.24	0.96	1.03	0.32
14:0	2.30	0.53	2.53	0.51	4.06	2.45	2.98	0.39
15:0	0.61^a^	0.25	0.60^a^	0.41	1.67^b^	0.71	1.12^b^	0.15
16:0	30.74	2.17	29.09	3.62	28.18	3.48	30.74	1.62
18:0	2.14^a^	0.31	2.20^a^	0.70	9.05^b^	5.53	2.71^a^	1.00
20:0	0.37	0.08	0.46	0.22	1.04	1.58	0.67	0.23
Total SFA	40.62	2.50	40.55	7.46	54.29	21.06	44.25	3.07
**PUFA**								
18:1	35.45^a^	1.93	29.51^b^	4.05	22.94^c^	4.48	24.01^c^	2.90
18:2	19.24	2.11	19.30	4.48	15.91	4.92	19.70	2.25
18:3*n*-3	1.65^a^	0.24	7.85^b^	1.22	7.25^b^	1.60	8.88^b^	1.10
20:4	1.61^a^	0.27	1.11^b^	0.29	1.3^b^	0.24	1.09^b^	0.12
20:5	0.08^a^	0.02	0.18^b^	0.04	0.26^c^	0.06	0.21^b,c^	0.06
22:5*n*-3	0.85	0.26	1.01	0.23	5.35	9.32	1.41	0.41
22:6	0.31^a^	0.07	0.34^a^	0.07	0.53^b^	0.18	0.45^b^	0.12
Total hydroperoxides	0.09	0.01	0.08	0.02	0.08	0.02	0.07	0.01
CD 18:3 (CNLA)	0.02^a^	0.01	0.02^a^	0.005	0.04^b^	0.02	0.03^b^	0.01
CLA 9,11	0.08^a^	0.02	0.07^a^	0.01	0.10^b^	0.04	0.15^b^	0.07

SFA, saturated fatty acid; PUFA, polyunsaturated fatty acid; CD, conjugated diene; CNLA, conjugated α-linolenic acid; CLA, conjugated linoleic acid. ^a,b,c^Mean values within a row with unlike superscript letters were significantly different (*P* < 0.05 ANOVA followed by *post hoc* Tukey’s multiple comparison test).

**Table 4:** Primer sequences used for real-time PCR.

Liver Gene Symbol	Forward Primer	Reverse Primer
β-actin	5′-AGAGGGAAATCGTGCGTGAC-3′	5′-CAATAGTGATGACCTGGCGT-3′
Δ-6-desaturase	5′-CCTGGACCGTGGCAAAAG-3′	5′-GTGGGACAGGAGGAGAAAGAAG-3′
Δ-5-desaturase	5′-CACTGTGGCCTTCTCTTCTCA-3′	5′-ACGCGGTTTTCTTATCTGTCA-3′
ELOVL-2	5′-AGCTGCCATGCCCTTTCTGA-3′	5′-CCCTGGGGCTCTGTTGATTATG-3′
ELOVL-5	5′-GTCCTCCATCCCGTCCAT-3′	5′-TGATTGTCAGCACAAACTGGA-3′
ELOVL-6	5′-TGGCTGGCTTGAAAATGGAGTCTT-3′	5′-GAACAGGGAGGGAGGCGAACAC-3′
SCD-1	5′-ACTGCTGGGGCGAGACTTTTGTA-3′	5′-CCGGGATTGAATGTTCTTGTCGTA-3′
CD-36	5′-TGATACTATGCCCGCCTCTCC-3′	5′-TTTCCCACACTCCTTTCTCCTCTA-3′
FAS	5′-TCCACCTTTAAGTTGCCCTG-3′	5′-TCTGCTCTCGTCATGTCACC-3′
SREBP-1c	5′-CTCCAGCTCATCAACAACCAAGAC-3′	5′-AGAGGAGGCCAGAGAAGCAGAAGA-3′
FATP-5	5′-CCGGCAGCATGGCGTAACAG-3′	5′-ACACATTTGCCCGAAGTCCATTG-3′
FABP-1	5′-GAAGCCTCGTTGCCACCAT-3′	5′-CGATTTCTGACACCCCCTTGAT-3′
**Ileum Gene Symbol**	**Forward Primer**	**Reverse Primer**
FABP-2	5′-TGAGGCCAAGCGATTCT-3′	5′-TGAGCCTGGCATTAGCAT-3′
FATP-4	5′-TGCCCGCCCCATCTTCCT-3′	5′-AACAGCGGGTCTTTCACAACAG-3′
DGAT-2	5′-CACAGGTGCCGTCTTGGGTTATC-3′	5′-CAGACTTGGGGTGTGGCTCAGGA-3′
