# Supplementary material for: Bifidobacterium breve with α-linolenic acid alters the composition, distribution and transcription factor activity associated with metabolism and absorption of fat
Source: Sci Rep. 2017 Mar 7;7:43300. doi: 10.1038/srep43300 (PMC5339701; doi:10.1038/srep43300)
Supplement: SUPPLEMENTARY TABLE 1 [file srep43300-s1.pdf]

Bifidobacterium breve with α-linolenic acid alters the composition, distribution and transcription factor activity associated with metabolism and absorption of fat.

Elaine Patterson[1,2], Rebecca Wall[1,2], Sara Lisai[3], R. Paul Ross[1], Timothy G. Dinan[1,4], John F. Cryan[1,5], Gerald F. Fitzgerald[1,6], Sebastiano Banni[3], Eamonn M. Quigley[1], Fergus Shanahan[1], and Catherine Stanton[1,2*].

[1]APC Microbiome Institute, Biosciences Building, University College Cork, Cork, Ireland

[2]Teagasc Food Research Centre, Food Biosciences Department, Moorepark, Fermoy, Cork, Ireland

[3]Department of Biomedical Sciences, University of Cagliari, Monserrato, CA 09042, Italy

[4]Department of Psychiatry and Neurobehavioural Science, University College Cork, Cork, Ireland

[5]Department of Anatomy and Neuroscience, University College Cork, Cork, Ireland

[6]School of Microbiology, University College Cork, Cork, Ireland

*Corresponding author:

Prof. Catherine Stanton,

Teagasc Food Research Centre,

Food Biosciences Department,

Moorepark, Fermoy, Cork, Ireland.

E-mail: catherine.stanton@teagasc.ie

Tel: +353-25-42606

SUPPLEMENTARY TABLE 1 Endocannabinoid levels (%mol) in the liver and epididymal adipose tissue of mice fed an ALA enriched diet either alone or in combination with *Bifidobacterium breve* NCIMB 702258 or *B. breve* DPC 6330 or an unsupplemented diet for 6 weeks.

**Endocannabinoids and Congeners**	**CON** *n = 9*		**ALA-CON** *n = 9*		**ALA+ NCIMB 702258** *n = 8*		**ALA+ DPC 6330** *n = 9*	
	mol % * 1000		***mol % * 1000***		***mol % * 1000***		***mol % * 1000***	
**Liver**	**Mean**	**SD**	**Mean**	**SD**	**Mean**	**SD**	**Mean**	**SD**
AEA	2.9[a]	0.87	1.64[b]	0.39	0.65[c]	0.21	1.09[c]	0.47
PEA	12.58[a]	4.89	32.2[b]	15.58	7.37[c]	2.29	7.52[c]	3.12
OEA	12.66[a]	3.00	7.39[b]	2.57	5.63[b]	1.41	3.68[c]	0.99
2AG	74.07[a]	23.45	30.18[b]	19.29	20.31[b]	9.69	24.62[b]	12.67
DHEA	3.12	2.47	1.54	1.18	0.67	0.37	2.50	1.97
**Epididymal Adipose Tissue**								
AEA	2.72[a]	0.40	2.34[a]	0.65	7.94[b]	1.33	7.27[b]	2.60
PEA	0.46[a]	0.18	0.38[a]	0.12	1.16[b]	0.51	0.51[a]	0.17
OEA	1.00[a]	0.41	0.99[a]	0.35	1.87[b]	0.82	1.02[a]	0.47
2AG	3.84	1.77	2.42	1.37	6.35	4.86	3.66	2.72
DHEA	0.16[a]	0.02	0.13[a]	0.06	0.40[b]	0.24	0.25[b]	0.09

AEA, N-arachidonoylethanolamide; PEA, N-palmitoylethanolamide; OEA, oleoylethanolamide; 2AG, 2-arachidonoylglycerol; DHEA, docosahexaenoylethanolamide. [a,b,c] Mean values within a row with unlike superscript letters were significantly different ($P <$ 0.05 ANOVA followed by *post hoc* Tukey's multiple comparison test).
